# Effectiveness of an mHealth Intervention Combining a Smartphone App and Smart Band on Body Composition in an Overweight and Obese Population: Randomized Controlled Trial (EVIDENT 3 Study)

**DOI:** 10.2196/21771

**Published:** 2020-11-26

**Authors:** Cristina Lugones-Sanchez, Maria Antonia Sanchez-Calavera, Irene Repiso-Gento, Esther G Adalia, J Ignacio Ramirez-Manent, Cristina Agudo-Conde, Emiliano Rodriguez-Sanchez, Manuel Angel Gomez-Marcos, Jose I Recio-Rodriguez, Luis Garcia-Ortiz, EVIDENT 3 Investigators

**Affiliations:** 1 Institute of Biomedical Research of Salamanca (IBSAL) Primary Care Research Unit of Salamanca (APISAL) Health Service of Castilla y León (SACyL) Salamanca Spain; 2 Institute for Health Research Aragón (IISA) Department of Internal Medicine, Psychiatry and Dermatology, University of Zaragoza Zaragoza Spain; 3 Valladolid Rural Health Center I. Health Service of Castilla y León (SACyL) Valladolid Spain; 4 University of Castilla-La Mancha, Health and Social Research Center Cuenca Spain; 5 Calvià Primary Care Center, Health Service of Balear Islands Balear Islands Spain; 6 Institute of Biomedical Research of Salamanca (IBSAL), Primary Care Research Unit of Salamanca (APISAL) Health Service of Castilla y León (SACyL) Department of Medicine, University of Salamanca Salamanca Spain; 7 Institute of Biomedical Research of Salamanca (IBSAL), Primary Care Research Unit of Salamanca (APISAL) Health Service of Castilla y León (SACyL) Department of Nursing and Physiotherapy, University of Salamanca Salamanca Spain; 8 Institute of Biomedical Research of Salamanca (IBSAL), Primary Care Research Unit of Salamanca (APISAL) Health Service of Castilla y León (SACyL) Department of Biomedical and Diagnostic Sciences, University of Salamanca Salamanca Spain; 9 Spanish Research Network for Preventive Activities and Health Promotion in Primary Care (REDIAPP) Barcelona Spain

**Keywords:** diet records, mobile app, telemedicine, electric impedance, obesity, body fat distribution, weight control

## Abstract

**Background:**

Mobile health (mHealth) is currently among the supporting elements that may contribute to an improvement in health markers by helping people adopt healthier lifestyles. mHealth interventions have been widely reported to achieve greater weight loss than other approaches, but their effect on body composition remains unclear.

**Objective:**

This study aimed to assess the short-term (3 months) effectiveness of a mobile app and a smart band for losing weight and changing body composition in sedentary Spanish adults who are overweight or obese.

**Methods:**

A randomized controlled, multicenter clinical trial was conducted involving the participation of 440 subjects from primary care centers, with 231 subjects in the intervention group (IG; counselling with smartphone app and smart band) and 209 in the control group (CG; counselling only). Both groups were counselled about healthy diet and physical activity. For the 3-month intervention period, the IG was trained to use a smartphone app that involved self-monitoring and tailored feedback, as well as a smart band that recorded daily physical activity (Mi Band 2, Xiaomi). Body composition was measured using the InBody 230 bioimpedance device (InBody Co., Ltd), and physical activity was measured using the International Physical Activity Questionnaire.

**Results:**

The mHealth intervention produced a greater loss of body weight (–1.97 kg, 95% CI –2.39 to –1.54) relative to standard counselling at 3 months (–1.13 kg, 95% CI –1.56 to –0.69). Comparing groups, the IG achieved a weight loss of 0.84 kg more than the CG at 3 months. The IG showed a decrease in body fat mass (BFM; –1.84 kg, 95% CI –2.48 to –1.20), percentage of body fat (PBF; –1.22%, 95% CI –1.82% to 0.62%), and BMI (–0.77 kg/m^2^, 95% CI –0.96 to 0.57). No significant changes were observed in any of these parameters in men; among women, there was a significant decrease in BMI in the IG compared with the CG. When subjects were grouped according to baseline BMI, the overweight group experienced a change in BFM of –1.18 kg (95% CI –2.30 to –0.06) and BMI of –0.47 kg/m^2^ (95% CI –0.80 to –0.13), whereas the obese group only experienced a change in BMI of –0.53 kg/m^2^ (95% CI –0.86 to –0.19). When the data were analyzed according to physical activity, the moderate-vigorous physical activity group showed significant changes in BFM of –1.03 kg (95% CI –1.74 to –0.33), PBF of –0.76% (95% CI –1.32% to –0.20%), and BMI of –0.5 kg/m^2^ (95% CI –0.83 to –0.19).

**Conclusions:**

The results from this multicenter, randomized controlled clinical trial study show that compared with standard counselling alone, adding a self-reported app and a smart band obtained beneficial results in terms of weight loss and a reduction in BFM and PBF in female subjects with a BMI less than 30 kg/m^2^ and a moderate-vigorous physical activity level. Nevertheless, further studies are needed to ensure that this profile benefits more than others from this intervention and to investigate modifications of this intervention to achieve a global effect.

**Trial Registration:**

Clinicaltrials.gov NCT03175614; https://clinicaltrials.gov/ct2/show/NCT03175614.

**International Registered Report Identifier (IRRID):**

RR2-10.1097/MD.0000000000009633

## Introduction

Obesity and overweight are considered a major public health issue. The prevalence of obesity has reached epidemic levels, with 650 million adults worldwide estimated to be obese in 2016 [1]. Additionally, more than one-half of the population in Europe is classified as overweight or obese [2]. In the case of Spain, the proportion of obese people may reach 58% by 2030 [3]. The association of obesity with cardiovascular diseases and type 2 diabetes [4], among other diseases, is well known, and obesity also exacerbates cardiovascular risk factors [5]. Thus, it contributes to an increase in the mortality rate worldwide, as a more frequent cause than underweight or malnutrition [6]. This situation makes the development and implementation of weight management interventions imperative.

Because of the complex nature of obesity, a multifactorial strategy is needed. The modification of lifestyles is the cornerstone of weight management, including diverse aspects, such as reduced energy intake, increased energy expenditure, exercise, and behavior change strategies [7,8]. Primary care providers (PCPs) play a critical role in recommending appropriate weight-loss strategies. Moreover, the positive effect of PCP advice on patient engagement in weight loss efforts has been demonstrated [9]. Unfortunately, there are some barriers to obesity management, such as the lack of tools or training [10]. Also, interventions are usually individual and face-to-face, which generates more demands by the patient, thereby increasing the burden on health care professionals and the health care system.

Mobile health (mHealth) could be an excellent strategy for PCPs to implement with their patients to help them maintain lifestyle changes. Information and communication technologies (ICTs) have the potential to standardize and improve the quality of treatment provided and increase resources for prevention activities [11]. They also allow PCPs to address barriers through enhancing self-monitoring of the patient by registering progress or symptoms, which could improve feedback communication and enable PCPs to spend less time gathering routine data and more time engaging with patients. This means of interaction might enhance treatment outcomes as well as improve follow-up of some chronic diseases [12] while optimizing PCP time and reducing costs [13]. Every year, thousands of mobile apps are developed with the purpose of improving lifestyles. To ensure that these tools are able to have a positive influence, more studies are needed because most apps available are suboptimal in quality, meaning that they have inadequate scientific coverage and accuracy of weight-related information [14]. Compared with usual practice, the use of ICTs in the primary care context might help patients to achieve significant weight loss [15], including patients who are socioeconomically disadvantaged [16], thereby increasing egalitarian access to treatment. However, further research is needed to determine the optimal use of technology in weight loss, since the inclusion of small sample sizes, and the variability in study designs, follow-up times, and interventions, may hinder replication and comparison of results [17], leading to unclear conclusions in this regard.

In relation to weight loss interventions on body composition, some studies have reported the effect of an energy-restricted high-protein diet combined with exercise on decreasing fat-free mass [18] and leisure-time exercise in reducing fat mass [19]. In recent years, some studies have provided important findings related to the feasibility of ICT interventions in this practical setting, such as the LEAN study [20] and the IDEA study [21]. In addition, a recent pilot study assessing a telenutrition weight loss intervention in primary care showed greater loss of weight and body fat in obese men compared with usual care [22]. These results spotlight the need for more research in this field in order to achieve the optimal combination of health tools and the time needed to achieve changes.

However, previous studies have usually considered weight and BMI as the main outcomes. Although BMI is easy to obtain, it is an indirect measure of body composition and, therefore, less accurate than other measures [23] in estimating the distribution of body fat, resulting in misclassification of obesity. Recent studies have highlighted other useful measures involved in weight regulation, such as fat-free mass (FFM), body fat mass (BFM), and percentage of body fat (PBF), which could better explain body composition changes during weight interventions [24]. These variables are analyzed by bioelectrical impedance analysis (BIA), an indirect measure that uses multiple electrical currents through the body to estimate the percentage of different types of body tissue. Regarding the PBF, the correlation between BIA and the reference measure—dual-energy X-ray absorptiometry—was 0.88 for a healthy population [25], with a mean difference of –1.83 (SD 4.1%) for all subjects. In addition, BIA is the most cost-effective method of measuring body composition [26], making it a good alternative for its estimation.

Furthermore, it is important to determine whether these technologies can increase weight loss and modify body composition to clinically significant levels, which would show that ICTs could potentially be useful in tackling obesity. This study aims to assess the short-term effectiveness of a 3-month intervention that includes a smartphone app in combination with a smart band to lose weight and change body composition in sedentary Spanish adults who are overweight or obese.

## Methods

### Design and Scope

EVIDENT 3 is a randomized controlled, multicenter clinical trial with two parallel groups with a follow-up period of 12 months. The study was conducted in a primary care setting. The Primary Care Research Unit in Salamanca (APISAL) at the Biomedical Research Institute of Salamanca (IBSAL) coordinated the project in five primary care centers belonging to the Network for Preventive Activity and Health Promotion (REDIAPP) (Salamanca, Valladolid, Cuenca, Palma de Mallorca, and Zaragoza). Between June 2017 and November 2019, evaluations were made at baseline and at the 3-month follow-up visit. The results presented in this paper correspond to the short-term effect (3 months) of the intervention on body composition, considered one of the EVIDENT 3 study’s secondary outcomes.

### Study Population

The participants were selected by random sampling among the patients attending a consultation with their family doctor in each participating center. The inclusion criteria were age between 20 and 65 years, a BMI between 27.5 kg/m^2^ and 40 kg/m^2^, agreement to participate in the study, and signing the informed consent document. A detailed description of inclusion and exclusion criteria has been published in the study protocol [27]. To determine the effect of the intervention on body composition, an additional criterion was set: only subjects with both body composition measurements (at baseline and 3-month visit) assessed using the InBody 230 Body Composition Analyzer (InBody Co., Ltd) were included in the analysis. Hence, the study sample consisted of 440 subjects.

### Sample Size

The sample size calculation was performed for the primary study endpoint of weight loss. Accepting an α risk of 0.05 and a β risk of 0.20, with an SD of 12 kg, and estimating from subjects from the EVIDENT 2 study [28], it was determined that 592 subjects would be needed (296 per group) to detect a decrease in weight of 3 kg or more [29] in the intervention group (IG) versus the control group (CG), taking into consideration a 15% loss of subjects at follow-up. This effect size represented a 3% to 5% difference between the groups, which was expected to produce clinically relevant health benefits [30]. There were 440 participants who completed the 3-month visit (IG, n=231; CG, n=209). Taking into account the sample size and a common SD of weight difference of 3.27 kg, the poststudy power to detect the 0.839 kg weight loss difference found between groups as significant was 77%.

### Randomization

**Figure 1:**
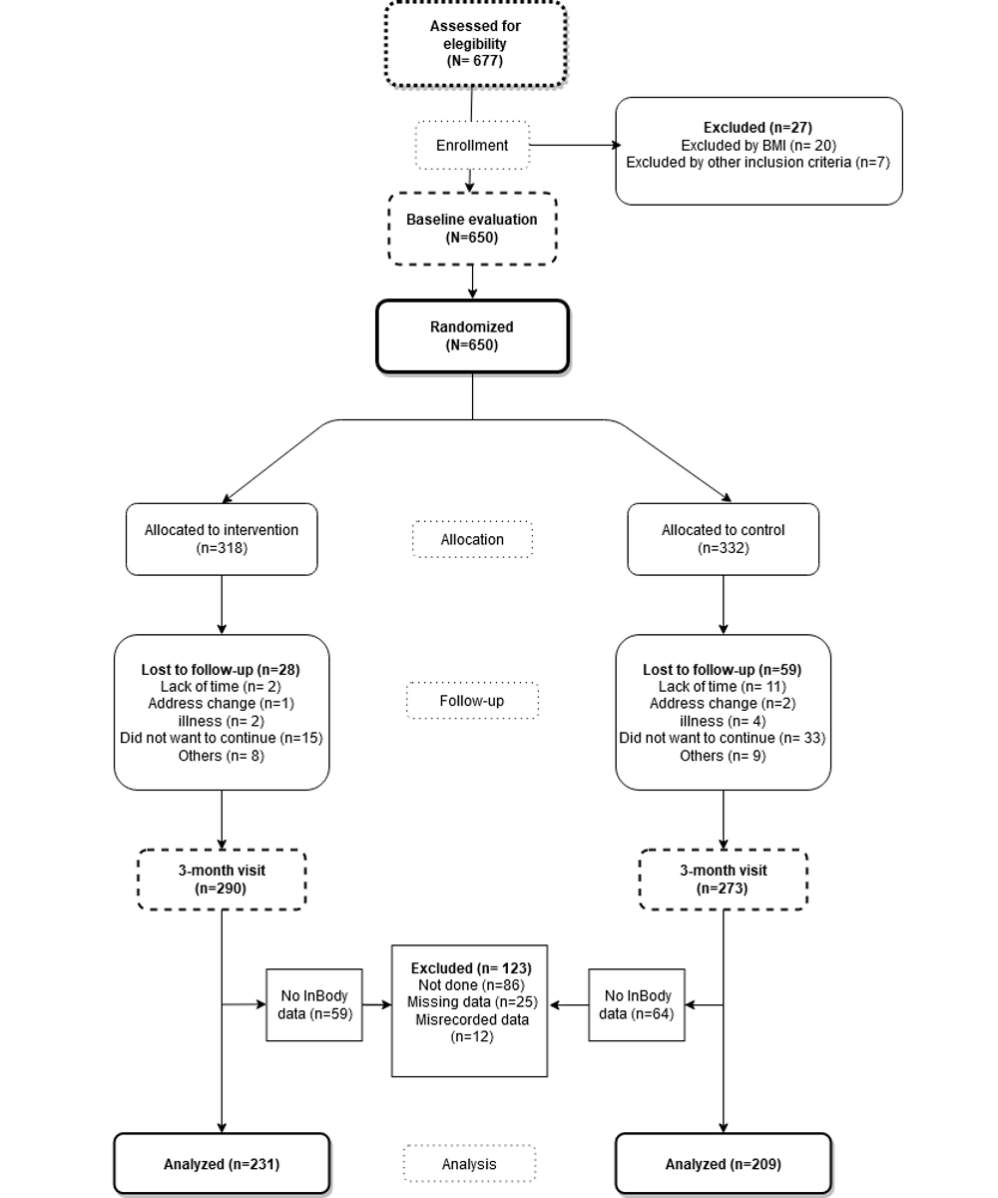
Flow chart depicting study enrolment and completion.

Participants were randomly assigned into two groups in a 1:1 ratio for the control group (CG) and intervention group (IG). Randomization was done after informed consent was obtained. The allocation sequence was generated through a standardized computer program (Epidat 4.2) by an independent researcher and concealed until the trial group was assigned (Figure 1). To minimize contamination between groups, the investigator who performed the intervention was different from the investigator who conducted the evaluation. The investigator who performed the data analysis was blinded to the subjects’ groups. Due to the nature of the study, the subjects could not be blinded to the intervention.

### Procedures

Each participant had to complete an initial visit and two follow-up visits, at 3 months and 12 months, after study inclusion. Baseline and follow-up data were collected by a research nurse. The IG completed an additional set of measurements at an appointment 7 days after baseline, with a different nurse performing the measurements, where the application was explained and the smart band given.

### Outcome Measurements

The primary outcome was weight loss. Secondary outcomes included changes in some parameters of body composition. All outcomes were measured at baseline and 3 months after randomization.

#### Weight Loss

Body weight was measured to the nearest 0.1 kg, with the subject barefoot, wearing light clothing, and removing heavy pocket items, using the portable InBody 230 Body Composition Analyzer (InBody Co., Ltd). Height was measured with the subject barefoot in a standing position using a portable scale and measurement system (Seca 222), and the average of 2 readings rounded to the nearest centimeter was recorded. BMI was calculated by dividing weight (in kg) by height squared (in m^2^). Following the recommendations of the Spanish Society for the Study of Obesity (SEEDO) [31], waist circumference was measured in duplicate, using a flexible tape parallel to the floor, at the level of the midpoint between the last rib and the iliac crest, with the subject standing without clothing, after inspiration. Hip circumference was similarly measured at the level of the trochanters.

#### Body Composition

Body composition was determined by multifrequency BIA using an InBody 230 analyzer, with tetra-polar 8-point tactile electrodes that estimate total body water (TBW), dry lean mass, BFM, skeletal muscle mass (SMM), PBF, distribution of lean body mass, ratio of segmental lean mass, basal metabolic rate (BMR), and impedance of each body segment. This validated device [32] uses multiple currents at varying frequencies to provide precise body composition analysis without empirical estimation, increasing the reliability of the results.

The measurement was taken in the morning, before noon, with the subject barefoot, wearing light clothing, and standing upright for approximately 5 minutes before testing, with at least 2 hours of fasting and an empty bladder. These recommendations aim to measure body composition to the highest accuracy possible. The standing patient was required to wipe the palms of the hands, thumbs, and soles of the feet with the InBody tissue before placing them in the electrodes properly before testing. Individuals with medical implant devices such as pacemakers, essential support devices, or orthopedic prostheses, as well as pregnant women, could not be tested.

#### Clinically Relevant Measures

Data on the sociodemographic characteristics of the population including age, sex, education level, occupation, smoking history, and personal history of hypertension, dyslipidemia, and diabetes mellitus, as well as any active medical treatment, were collected. Smoking history was assessed through questions about the participant’s smoking status (smoker or nonsmoker). We considered smokers to be subjects who currently smoked or who had stopped smoking less than 1 year before.

The short version of the International Physical Activity Questionnaire (IPAQ) [33] was used to measure activity subjectively. The IPAQ is a self-reported questionnaire that assesses physical activity performed at three intensity levels according to the energy expenditure estimated for each level: walking, moderate intensity, and vigorous intensity. For each level, participants reported frequencies such as days per week and average duration in minutes over the past week. This allowed the metabolic equivalents (METs) per minute per week to be calculated and subjects to be classified according to three activity levels: light, moderate, and vigorous.

Other variables were measured, including drug use, blood pressure, postprandial glucose, and biochemical parameters (total serum cholesterol, low-density lipoprotein-cholesterol, and high-density lipoprotein-cholesterol). A detailed description of how these variables were measured was published in the study protocol [27].

### Intervention

#### Standard Counselling (CG and IG)

Both groups (CG and IG) received 5 minutes of counselling at the end of the baseline visit and prior to randomization. A trained nurse at each primary health center, who was not involved in other aspects of the study, gave advice on physical activity and healthy diet according to the current international recommendations for the general population. The health benefits of physical activity and the recommendation to complete at least 30 minutes of moderate activity 5 days a week, or 20 minutes of vigorous activity 3 days a week, were explained. Counselling on food was in compliance with the plate method [34].

#### Specific Intervention (IG)

The IG received the smartphone app and a smart band (Mi Band 2, Xiaomi) for 3 months, corresponding to the length of the intervention. Once the baseline visit was completed, another 15-minute visit was carried out 7 days later, at which the subjects were trained to use the device and the app (EVIDENT 3 Application [record entry no.00/2017/2438]), which was specifically designed for the study by CGB Computer Company and APISAL.

**Figure 2:**
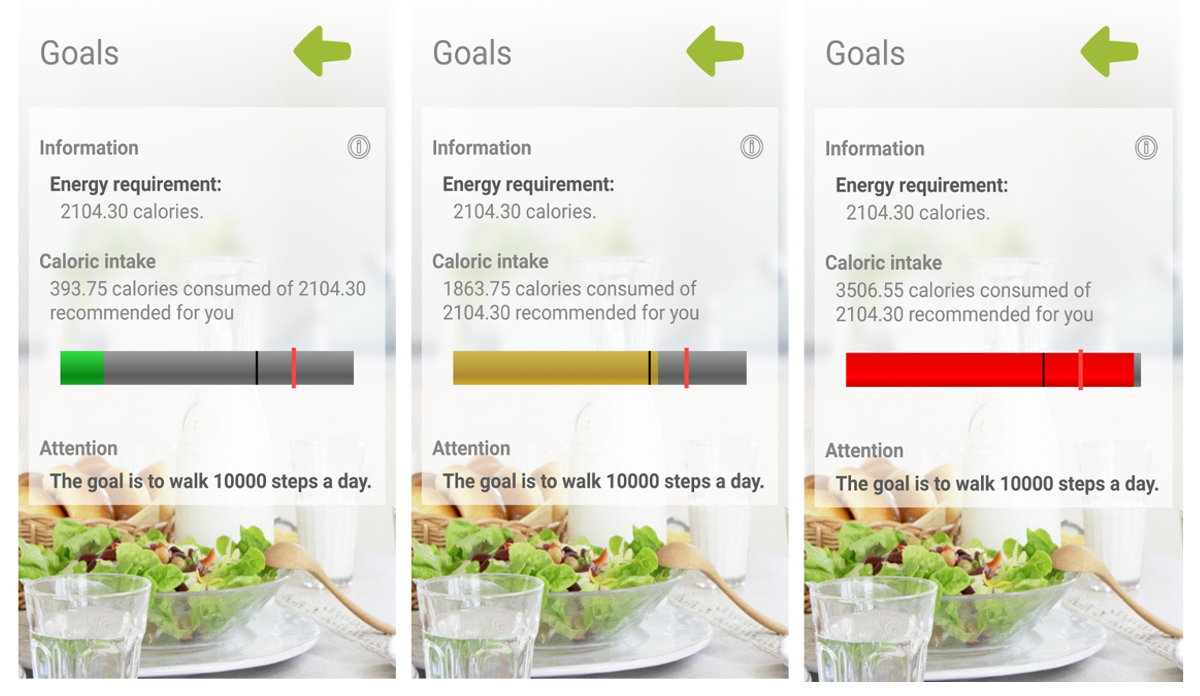
EVIDENT app feedback information.

During the 15-minute visit, the app was configured with each participant’s data (sex, age, weight, and height). It was designed to allow the dietary intake to be self-reported daily (Figure 2) and automatically record physical activity data from the smart band. Variables collected from the wearable device were number of steps taken, time of activity, kilometers traveled, and kilocalories expended. Subjects entered their food intake daily (divided into breakfast, midmorning snack, lunch, afternoon snack, and dinner) by selecting dishes and foods from the app menu and indicating the portion size. Thus, data collected comprised average energy intake (kcals), macronutrients and micronutrients (g/day), and time spent using the app (days). Food composition data were collected from the Spanish Food Composition Database [35] developed by RedBEDCA and AESAN. Once all of the daily information was collected, the app integrated the data to create personalized recommendations, based on the subjects’ characteristics, and specific objectives and goals for weight loss. The subject was able to consult the app for these recommendations, as well as information about caloric intake changes and macronutrient distribution. At the 3-month visit, the devices were collected. All information generated by the app was duly analyzed and entered into the database.

The behavioral strategies used in the intervention were those that enhance behavior changes toward healthier lifestyles. In this case, activities were meant to enhance self-efficacy, which is one of the most important determinants of behavior change [36,37], through self-monitoring, goal setting, and positive reinforcement. In order to avoid scheduling issues due to work shifts or other daily duties, participants were advised to use the app at the end of the day to register daily meals as well as to check physical activity information on the app. The smart band, worn at every moment, was set to congratulate the user when reaching 10,000 steps, following the general steps per day recommendation [38].

### Blinding Strategy

The investigator carrying out the intervention with the IG was different from the person responsible for the assessment and standard counselling; both were kept blinded throughout the study, as was the investigator conducting data analysis. Due to the nature of the study, the subjects could not be blinded. To prevent contamination between groups, in the assessment visits (at 3 and 12 months), only the study variables were evaluated, but no advice or reinforcement could be given. In addition, the app was not available for download on the internet until the end of the study, so the CG could not make use of it in any way. During the follow-up visits, participants were instructed not to use other digital health technologies.

### Ethical Considerations

The study was approved by the Clinical Research Ethics Committee of the Health Area of Salamanca on April 2016. All procedures were performed in accordance with the ethical standards of the institutional research committee and with the 2013 Declaration of Helsinki. All patients signed written informed consent documents prior to participation in the study. The trial was registered at ClinicalTrial.gov with identifier NCT03175614.

### Statistical Analysis

#### General Analysis

The results were expressed as mean (SD) for quantitative variables and as frequency distributions for qualitative variables. The statistical normality was tested using the Kolmogorov-Smirnov test. Chi-square and Fisher tests were used to analyze the association between independent qualitative variables. Student *t* and Mann-Whitney U tests were used for the comparison of means between 2 independent groups. Pearson correlation and Spearman rho were used to evaluate the relationship between quantitative variables.

#### Analysis of Intervention Effect on Primary and Secondary Outcomes

To analyze the changes at 3 months after baseline in primary (weight loss) and secondary endpoints within the same group, we used the paired *t* test or McNemar test for quantitative or dichotomous variables, respectively. To analyze the effect of the intervention, we performed a multivariate analysis of variance of repeated measures, adjusted by the baseline value of each variable, in the follow-up for primary and secondary endpoints.

#### Analysis by Subgroups

We carried out subanalyses of the intervention effect on primary and secondary outcomes by sex (men and women), BMI at baseline (<30 kg/m^2^ and ≥30 kg/m^2^), and initial self-reported physical activity level (light and moderate-vigorous physical activity). Subanalyses were sufficiently powered (>65%) to detect differences in women, moderate-vigorous physical activity, and overweight and obesity, but not in men or light physical activity.

The contrast in hypotheses established an α of .05. The data were analyzed using SPSS Statistics software (version 25.0; IBM Corp).

## Results

### Baseline Characteristics of the Participants and Follow-up

A total of 650 subjects fulfilled all of the inclusion criteria. They were included into the program and randomized to the IG or CG. Participant flow is presented in Figure 1. Testing at the 3-month visit was completed by 563 of 650 participants (86.6%).

In addition to the 87 subjects (13.4%) who dropped out during the study (Figure 1), 123 (123/650, 18.9%) subjects were excluded from the analysis. Exclusion requirements to perform the test were met in 86 subjects (86/650, 13.2%), so there were no measurements at any visit. In addition, the measurements of 37 subjects were not included due to incorrectly performed tests (25/650, 3.8%) or misrecorded data (12/650, 1.8%). Thus, 440 subjects (IG: n= 231; CG: n= 209) were included in the final analysis.

Both groups had a similar mean age—47.4 (SD 10.0) years in the IG and 48.8 (SD 9.2) years in the CG—and most participants were women (IG: 161/231, 69.7%; CG: 144/209, 68.9%) (Table 1). Most participants had middle or high school education or higher (206/231, 89.2% and 181/209, 86.6%) and a mean baseline BMI of 32.8 (SD 3.3) and 32.9 (SD 3.4) in the IG and CG, respectively. No differences in baseline characteristics were observed between the IG and CG.

**Figure 3:**
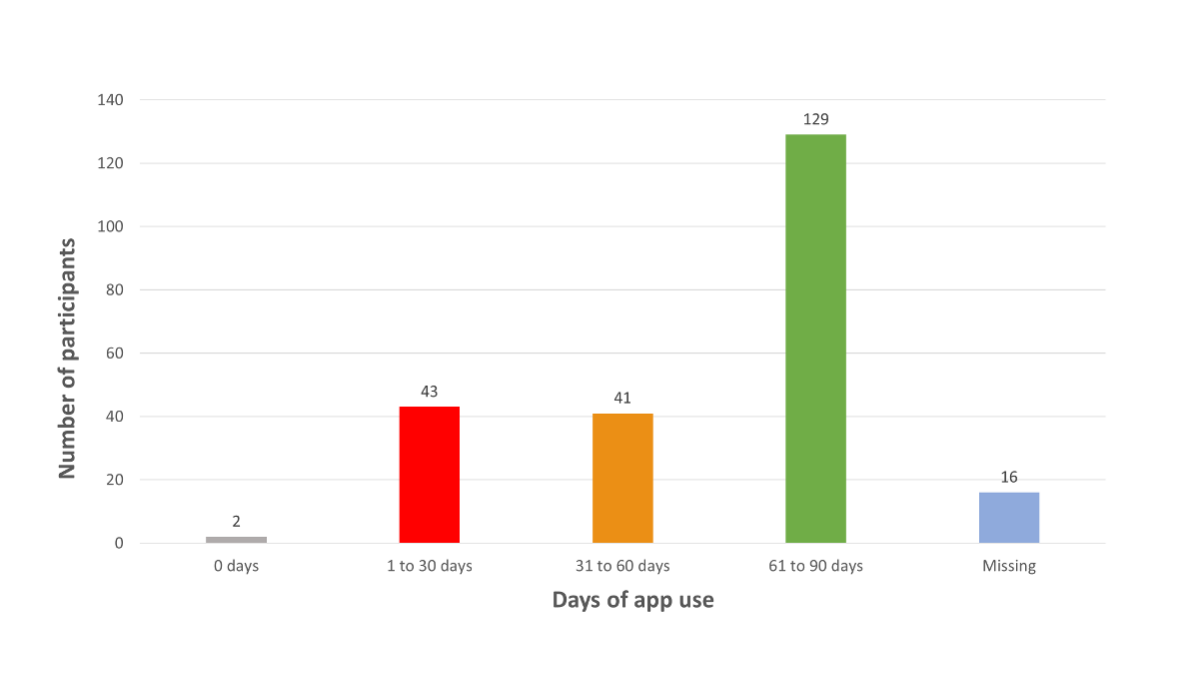
Adherence to the EVIDENT smartphone app (number of days with a record in the app).

Adherence to the smartphone app in the IG was calculated from app output data, showing that 129 of 231 (55.8%) subjects adhered sufficiently by using it for 60 days or more, 41 of 231 (17.8%) subjects used the app for 31 to 60 days, and 43 of 231 (18.6%) subjects entered data on 30 days or less. Two subjects (2/231, 0.9%) did not register any food intake information, and there were 16 corrupted files (16/231, 6.9%) from which no information was obtainable (Figure 3).

Regarding body composition variables, which were evaluated using BIA, no differences were found between the groups, with a mean weight of 89.7 kg (SD 13.1) in the IG and 90.7 kg (SD 13.9) in the CG. PBF, estimated using the InBody device, was 41.8% (SD 7.6%) and 42.1% (SD 6.4%) in the IG and CG, respectively. The main variables related to body composition are shown in Table 2.

In terms of self-reported physical activity, the IG had a total of 1263.6 METs/min/week and the CG had a total of 1353.3 METs/min/week, measured using the 7-day IPAQ, with no difference between them. At baseline, most of the sample showed a moderate physical activity level, in both the IG (50.2%) and the CG (51.2%).

**Table 1:** Baseline characteristics of the study population (N=440).

Baseline characteristics			Intervention group (n=231)	Control group (n=209)
Age (years), mean (SD)			47.4 (10.0)	48.8 (9.2)
Female sex, n (%)			161 (69.7)	144 (65.7)
**Work situation, n (%)**				
	Works outside the home		170 (73.6)	157 (75.1)
	Homemaker		20 (8.6)	14 (6.7)
	Retired		14 (6.1)	13 (6.2)
	Student		8 (3.5)	4 (1.9)
	Unemployed		19 (8.2)	21 (10.1)
**Educational level, n (%)**				
	University studies		97 (42.0)	88 (42.1)
	Middle or high school		109 (47.2)	93 (44.5)
	Elementary school		25 (10.8)	28 (13.4)
**Smoking status, n (%)**				
	Nonsmoker		97 (42.0)	90 (43.1)
	Smoker		46 (19.9)	44 (21.0)
	Former smoker		88 (38.1)	75 (35.9)
**Clinical variables, mean (SD)**				
		BMI (kg/m^2^)	32.8 (3.3)	32.9 (3.4)
		Waist circumference (cm)	105.9 (10.1)	107.1 (9.8)
		Systolic blood pressure (mmHg)	118.3 (14.4)	119.8 (15.5)
		Diastolic blood pressure (mmHg)	79.2 (8.7)	80.4 (9.9)
		Total cholesterol (mg/dL)	199.7 (35.6)	201.7 (41.4)
		Triglycerides (mg/dL)	127.3 (73.5)	127.3 (63.3)
		Fasting plasma glucose (mg/dL)	92.5 (12.6)	94.3 (15.7)
		Glycated hemoglobin (%)	5.4 (0.4)	5.5 (0.4)
**Chronic diseases, n (%)**				
		Hypertension	57 (24.7)	72 (34.5)
		Dyslipidemia	59 (25.5)	61 (29.2)
		Diabetes	8 (3.5)	9 (4.3)
**Medication use, n (%)**				
		Antihypertensive drugs	35 (15.2)	41 (19.6)
		Lipid-lowering drugs	40 (17.3)	39 (18.7)
**BMI classification, n (%)**				
		BMI≤30	60 (26.0)	50 (23.9)
		BMI>30	171 (74.0)	159 (76.1)
**Physical activity classification, n (%)**				
		Light physical activity	93 (40.3)	86 (41.2)
		Moderate physical activity	116 (50.2)	107 (51.2)
		Vigorous physical activity	22 (9.5)	16 (7.6)

**Table 2:** Anthropometric and physical activity baseline data.

Variables		Intervention group (n=231), mean (SD)	Control group (n=209), mean (SD)	*P* value^a^
**Body composition**				
	Weight (kg)	89.7 (13.1)	90.7 (13.9)	.43
	Total body water (kg)	38.4 (7.9)	38.6 (7.8)	.73
	Protein (kg)	10.3 (2.2)	10.4 (2.1)	.76
	Minerals (kg)	3.6 (0.7)	3.7 (0.7)	.72
	Body fat mass (kg)	37.4 (8.5)	38.1 (7.9)	.39
	Fat-free mass (kg)	52.3 (10.8)	52.6 (10.6)	.74
	Skeletal muscle mass (kg)	29.1 (6.6)	29.2 (6.4)	.77
	BMI (kg/m^2^)	32.7 (3.3)	32.9 (3.4)	.62
	Body fat (%)	41.8 (7.6)	42.1 (6.4)	.68
	Basal metabolic rate (kcal/day)	1499.4 (233.0)	1506.8 (229.3)	.74
	Waist-to-hip ratio	1.0 (0.1)	1.0 (0.1)	.18
**Physical activity**				
	METs^b^ of intense activity	164.5 (258.9)	677.9 (1333.8)	.34
	METs of moderate activity	218.9 (141.2)	656.6 (587.8)	.19
	METs of light activity	880.1 (884.8)	901.6 (968.0)	.96
	Total METs/minute/week	1263.6 (1285.0)	1353.3 (1723.2)	.88

^a^*P* value differences between intervention group and control group.

^b^METs: metabolic equivalents.

### Changes in Body Weight During Study Period

The IG showed large changes in body weight (–1.97 kg, 95% CI –2.39 to –1.54) between baseline and 3 months, while the change was smaller (–1.13 kg, 95% CI –1.56 to –0.69) in the CG. Comparing groups, the IG achieved a weight loss of 0.84 kg more than the CG at 3 months (Table 3).

Analyzing by sex, there were no significant changes observed in body weight among men in the CG and IG. However, women in the IG had a significant weight loss of 1.37 kg (95% CI –2.03 to –0.71) compared with their CG counterparts.

**Table 3:** Effect of intervention on body composition variables for the total sample and by sex.

		Difference at 3 months		
Body composition variables		Intervention group (IG), mean (95% CI)	Control group (CG), mean (95% CI)	IG-CG, mean (95% CI)
**All subjects**				
	Weight (kg)	–1.97 (–2.39 to –1.54)*	–1.13 (–1.56, –0.69)*	–0.84 (–1.45 to –0.23)*
	TBW^a^ (kg)	–0.04 (–0.42 to 0.34)	–0.01 (–0.39 to 0.37)	–0.03 (–0.57 to 0.50)
	Protein (kg)	–0.04 (–0.17 to 0.08)	0.00 (–0.11 to 0.11)	–0.04 (–0.21 to 0.12)
	Minerals (kg)	–0.04 (–0.11 to 0.03)	–0.01 (–0.04 to 0.02)	–0.03 (–0.11 to 0.05)
	BFM^b^ (kg)	–1.84 (–2.48 to –1.20)*	–1.11 (–1.69 to –0.53)*	–0.73 (–1.59 to 0.14)
	FFM^c^ (kg)	–0.13 (–0.63 to 0.38)	–0.01 (–0.53 to 0.50)	–0.11 (–0.83 to 0.60)
	SMM^d^ (kg)	–0.12 (–0.49 to 0.25)	0.02 (–0.31 to 0.35)	–0.14 (–0.64 to 0.36)
	BMI (kg/m^2^)	–0.77 (–0.96 to –0.57)*	–0.23 (–0.46 to 0.01)	–0.54 (–0.84 to –0.24)*
	PBF^e^ (%)	–1.22 (–1.82 to –0.62)*	–0.79 (–1.34 to –0.25)*	–0.42 (–1.24 to 0.39)
	BMR^f^ (kcal/day)	–2.63 (–13.49 to 8.23)	–0.30 (–11.37 to 10.77)	–2.34 (–17.82 to 13.15)
	WHR^g^	–0.03 (–0.07 to 0.01)	–0.01 (–0.01 to 0.00)*	–0.02 (–0.07 to 0.02)
**Men**				
	Weight (kg)	–1.70 (–2.54 to –0.85)*	–2.02 (–3.03 to –1.02)*	0.33 (–0.97 to 1.62)
	TBW (kg)	0.06 (–0.37 to 0.50)	–0.17 (0.55 to 0.20)	0.24 (–0.33 to 0.81)
	Protein (kg)	0.02 (–0.11 to 0.15)	–0.05 (–0.16 to 0.06)	0.07 (–0.10 to 0.24)
	Minerals (kg)	0.01 (–0.01 to 0.04)	–0.02 (–0.06 to 0.03)	0.03 (–0.02 to 0.08)
	BFM (kg)	–1.80 (–2.77 to –0.83)*	–1.79 (–2.51 to –1.07)*	–0.01 (–1.22 to 1.20)
	FFM (kg)	0.10 (–0.47 to 0.68)	–0.23 (–0.74 to 0.27)	0.34 (–0.43 to 1.11)
	SMM (kg)	0.11 (–0.27 to 0.49)	–0.11 (–0.43 to 0.20)	0.22 (–0.27 to 0.71)
	BMI (kg/m^2^)	–0.58 (–0.87 to –0.29)*	–0.55 (–0.92 to –0.18)*	–0.03 (–0.49 to 0.43)
	PBF (%)	–1.48 (–2.39 to –0.57)*	–1.14 (–1.60 to –0.68)*	–0.34 (–1.37 to 0.69)
	BMR (kcal/day)	2.35 (–10.11 to 14.81)	–5.05 (–15.98 to 5.89)	7.40 (–9.13 to 23.93)
	WHR	–0.01 (–0.02 to 0.00)**	–0.01 (–0.03 to 0.00)*	0.00 (–0.01 to 0.02)
**Women**				
	Weight (kg)	–2.08 (–2.58 to –1.59)*	–0.71 (–1.14 to –0.28)*	–1.37 (–2.03 to –0.71)*
	TBW (kg)	–0.09 (–0.61 to 0.43)	0.07 (–0.46 to 0.60)	–0.16 (–0.89 to 0.58)
	Protein (kg)	–0.07 (–0.24 to 0.10)	0.02 (–0.13 to 0.17)	–0.09 (–0.32 to 0.14)
	Minerals (kg)	–0.06 (–0.17 to 0.04)	0.00 (–0.04 to 0.04)	–0.06 (–0.18 to 0.06)
	BFM (kg)	–1.86 (–2.68 to –1.04)*	–0.80 (–1.58 to –0.02)**	–1.06 (–2.19 to 0.08)
	FFM (kg)	–0.23 (–0.91 to 0.45)	0.09 (–0.63 to 0.80)	–0.32 (–1.30 to 0.67)
	SMM (kg)	–0.22 (–0.73 to 0.29)	0.08 (–0.38 to 0.54)	–0.30 (–0.99 to 0.39)
	BMI (kg/m^2^)	–0.89 (–1.13 to –0.66)*	–0.17 (–0.39 to 0.05)	–0.72 (–1.05 to –0.40)*
	PBF (%)	–1.10 (–1.87 to –0.33)*	–0.63 (–1.41 to 0.14)	–0.47 (–1.56 to 0.62)
	BMR (kcal/day)	–4.84 (–19.59 to 9.90)	1.90 (–13.55 to 17.34)	–6.74 (–28.02 to 14.54)
	WHR	–0.04 (–0.10 to 0.03)	–0.01 (–0.01 to 0.00)	–0.03 (–0.10 to 0.04)

^a^TBW: total body water.

^b^BFM: body fat mass.

^c^FFM: fat-free mass.

^d^SMM: skeletal muscle mass.

^e^PBF: percentage of body fat.

^f^BMR: basal metabolic rate.

^g^WHR: waist-to-hip ratio.

*P<0.01.

**P<0.05.

### Changes in Body Composition After Intervention

**Figure 4:**
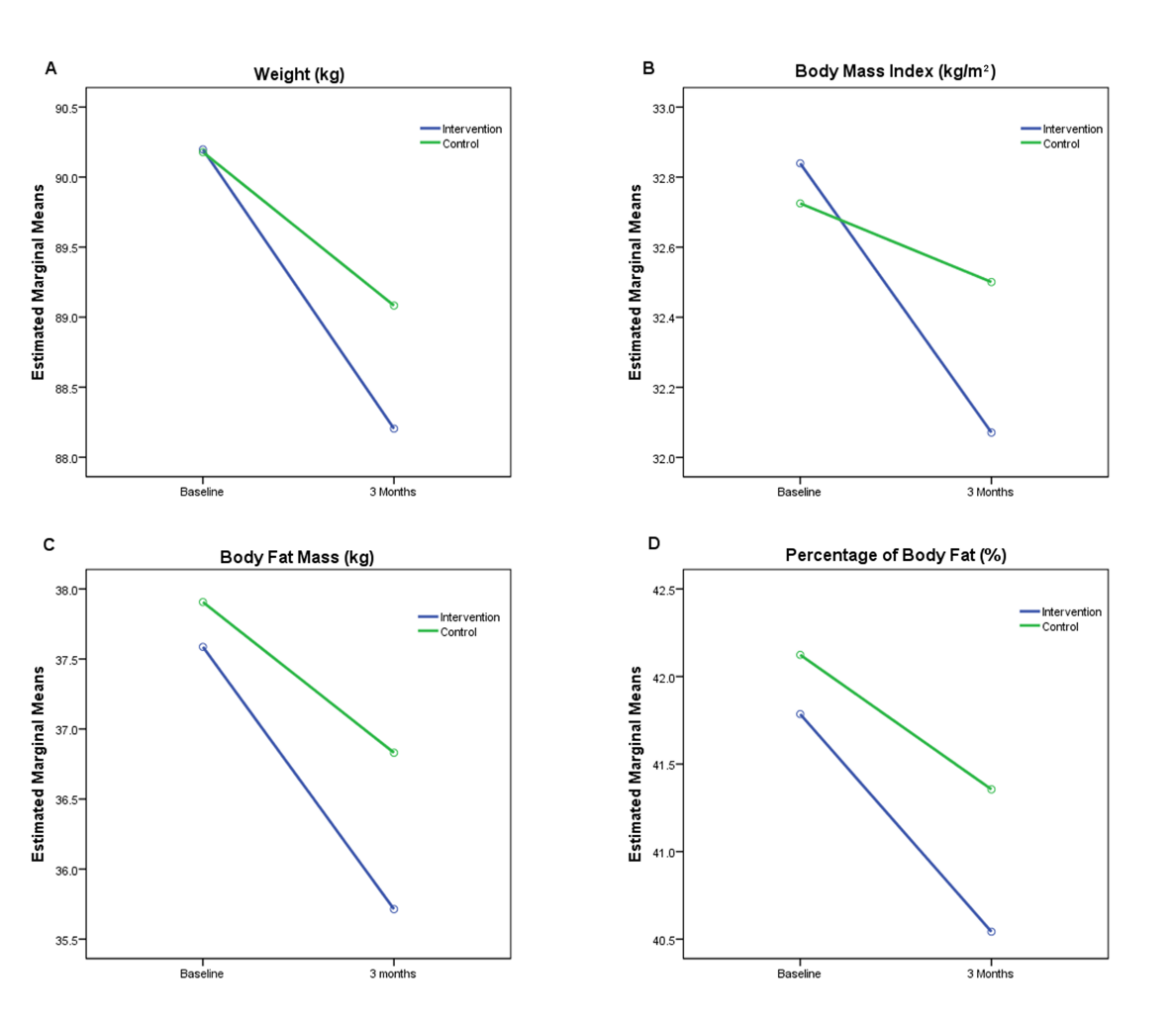
Evolution over time of main body composition variables. (A) Weight. (B) BMI. (C) Body fat mass. (D) Percentage of body fat.

The IG showed a decrease in body composition variables (Figure 4), with a change of –1.84 kg (95% CI –2.48 to –1.20) in BFM, –1.22% (95% CI –0.96% to –0.57%) in PBF, and 0.77 kg/m^2^ (95% CI –0.96 to –0.57) in BMI. A significant between-group difference was noted only in BMI (–0.54 kg/m^2^, 95% CI –0.84 to –0.24).

Analyzing by sex, men who received the intervention reduced BFM (–1.80 kg, 95% CI –2.77 to –0.83), PBF (–1.48%, 95% CI –2.39% to –0.57%), and BMI (–0.58 kg/m^2^, 95% CI –0.87 to –0.29). Women and men in the IG achieved similar results, decreasing BFM (–1.86 kg, 95% CI –2.68 to –1.04), PBF (–0.89%, 95% CI –1.13% to –0.66%), and BMI (–0.89 kg/m^2^, 95% CI –1.13 to –0.66). Although no significant changes were observed in any of these parameters in men, BMI was significantly reduced in women in the IG compared with women in the CG.

### Changes in Weight and Body Composition by Baseline BMI Classification

Regarding BMI groups at baseline (Table 4), weight loss in the IG was greater in subjects with type I obesity (BMI≥30 kg/m^2^) than in overweight (BMI<30 kg/m^2^) subjects, with significant results in both cases, although compared with the CG, the differences were higher in the overweight group (–1.10 kg, 95% CI –2.01 to –0.18) than in the group with type I obesity (–0.77 kg, 95% CI –1.52 to –0.01).

**Table 4:** Effect of intervention on body composition according to BMI.

		Difference at 3 months		
		Intervention group (IG), mean (95% CI)	Control group (CG), mean (95% CI)	IG-CC, mean (95% CI)
**BMI<30 kg/m^2^**				
	Weight (kg)	–1.76 (–2.42 to –1.11)*	–0.67 (–1.31 to –0.03)**	–1.10 (–2.01 to –0.18)**
	TBW^a^ (kg)	0.31 (–0.16 to 0.77)	0.24 (–0.04 to 0.53)	0.06 (–0.51 to 0.63)
	Protein (kg)	0.09 (–0.05 to 0.23)	0.06 (–0.02 to 0.14)	0.02 (–0.14 to 0.19)
	Minerals (kg)	0.02 (–0.01 to 0.05)	0.02 (–0.01 to 0.04)	0.00 (–0.03 to 0.04)
	BFM^b^ (kg)	–2.17 (–3.10 to –1.25)*	–0.99 (–1.55 to –0.43)*	–1.18 (–2.30 to –0.06)**
	FFM^c^ (kg)	0.41 (–0.21 to 1.04)	0.33 (–0.06 to 0.71)	0.09 (–0.67 to 0.85)
	SMM^d^ (kg)	0.28 (–0.12 to 0.69)	0.21 (–0.02 to 0.44)	0.07 (–0.42 to 0.56)
	BMI (kg/m^2^)	–0.68 (–0.92 to –0.44)*	–0.22 (–0.46 to 0.02)	–0.47 (–0.80 to –0.13)*
	PBF^e^ (%)	–2.01 (–3.02 to –0.99)*	–0.99 (–1.49 to –0.49)*	–1.01 (–2.21 to 0.18)
	BMR^f^ (kcal/day)	8.95 (–4.46 to 22.36)	7.02 (–1.31 to 15.35)	1.93 (–14.45 to 18.31)
	WHR^g^	–0.01 (–0.03 to 0.00)**	–0.01 (–0.01 to 0.00)**	–0.01 (–0.02 to 0.01)
**BMI≥30 kg/m^2^**				
	Weight (kg)	–2.04 (–2.57 to –1.50)*	–1.27 (–1.81 to –0.73)*	–0.77 (–1.52 to –0.01)**
	TBW (kg)	–0.16 (–0.65 to 0.33)	–0.09 (–0.58 to 0.40)	–0.07 (–0.77 to 0.62)
	Protein (kg)	–0.09 (–0.25 to 0.07)	–0.02 (–0.16 to 0.12)	–0.07 (–0.28 to 0.15)
	Minerals (kg)	–0.06 (–0.16 to 0.04)	–0.01 (–0.05 to 0.02)	–0.05 (–0.15 to 0.06)
	BFM (kg)	–1.72 (–2.52 to –0.92)*	–1.15 (–1.89 to –0.41)*	–0.57 (–1.67 to 0.52)
	FFM (kg)	–0.32 (–0.96 to 0.33)	–0.12 (–0.78 to 0.54)	–0.20 (–1.12 to 0.73)
	SMM (kg)	–0.26 (–0.74 to 0.22)	–0.04 (–0.47 to 0.38)	–0.22 (–0.86 to 0.43)
	BMI (kg/m^2^)	–0.84 (–1.07 to –0.60)*	–0.31 (–0.55 to –0.07)**	–0.53 (–0.86 to –0.19)**
	PBF (%)	–0.94 (–1.67 to –0.22)**	–0.73 (–1.44 to –0.03)**	–0.21 (–1.22 to 0.80)
	BMR (kcal/day)	–6.70 (–20.61 to 7.22)	–2.60 (–16.94 to 11.75)	–4.10 (–24.01 to 15.81)
	WHR	–0.04 (–0.09 to 0.02)	–0.01 (–0.02 to 0.00)**	–0.03 (–0.09 to 0.04)

^a^TBW: total body water.

^b^BFM: body fat mass.

^c^FFM: fat-free mass.

^d^SMM: skeletal muscle mass.

^e^PBF: percentage of body fat.

^f^BMR: basal metabolic rate.

^g^WHR: waist-to-hip ratio.

*P<0.01.

**P<0.05.

In terms of body composition variables, both the CG and the IG showed reductions in BFM, PBF, and waist-to-hip ratio (WHR), with the reductions being greater in the IG. Comparing these groups, the biggest reductions were seen in the overweight group, with reductions in BFM (–1.18 kg, 95% CI –2.30 to –0.06) and BMI (–0.47 kg/m^2^, 95% CI –0.80 to –0.13), whereas the group with type I obesity only decreased BMI (–0.53 kg/m^2^, 95% CI –0.86 to –0.19). We observed no significant between-group differences in other study variables.

### Changes in Weight and Body Composition by Baseline Self-Reported Physical Activity

When the data were analyzed according to the baseline physical activity level measured using the IPAQ (Table 5), there was a decrease in body composition variables in both the light physical activity and the moderate-vigorous physical activity groups within the IG. Participants in the light physical activity group lost similar weight (–1.99 kg, 95% CI –2.74 to –1.24) as those in the moderate-vigorous physical activity group (–1.95 kg, 95% CI –2.46 to –1.43). However, only the moderate-vigorous physical activity group achieved a significant net loss compared with the CG (–1.03 kg, 95% CI –1.76 to –0.29).

In addition, the IG decreased body composition variables, showing reductions in the moderate-vigorous physical activity group such as –1.89 kg (95% CI –2.36 to –1.42) in BFM, –1.34% (95% CI –1.70% to –0.97%) in PBF, and –0.76 kg/m^2^ (95% CI –0.95 to –0.57) in BMI, whereas the light physical activity group showed reductions of –1.77 kg (95% CI –3.21 to –0.33) in BFM and –0.85 kg/m^2^ (95% CI –1.21 to –0.49) in BMI. Comparing these results with their counterparts in the CG, we found that only BMI (–0.51 kg/m^2^, 95% CI –0.97 to –0.06) showed a significant difference in the light physical activity group, while the moderate-vigorous physical activity group reduced BFM (–1.03 kg, 95% CI –1.74 to –0.33), PBF (–0.76%, 95% CI –1.32% to –0.20%), and BMI (–0.51 kg/m^2^, 95% CI –0.83 to –0.19) significantly. Differences in other body composition variables were not found.

**Table 5:** Effect of intervention on body composition according to physical activity at baseline.

		Difference at 3 months					
		Intervention group (IG), mean (95% CI)		Control group (CG), mean (95% CI)		IG-CC, mean (95% CI)	
**LPA^a^**							
	Weight (kg)	–1.99 (–2.74 to –1.24)*		–1.42 (–2.17 to –0.66)*		–0.57 (–1.63 to 0.49)	
	TBW^b^ (kg)	–0.02 (–0.93 to 0.89)		0.06 (–0.76 to 0.89)		–0.08 (–1.31 to 1.15)	
	Proteins (kg)	–0.09 (–0.39 to 0.20)		0.01 (–0.23 to 0.25)		–0.10 (–0.49 to 0.28)	
	Minerals (kg)	–0.10 (–0.28 to 0.07)		–0.02 (–0.07 to 0.04)		–0.09 (–0.28 to 0.10)	
	BFM^c^ (kg)	–1.77 (–3.21 to –0.33)**		–1.48 (–2.67 to –0.29)**		–0.29 (–2.16 to 1.59)	
	FFM^d^ (kg)	–0.23 (–1.42 to 0.97)		0.06 (–1.05 to 1.17)		–0.29 (–1.92 to 1.34)	
	SMM^e^ (kg)	–0.29 (–1.19 to 0.60)		0.07 (–0.66 to 0.79)		–0.36 (–1.52 to 0.80)	
	BMI (kg/m^2^)	–0.85 (–1.21 to –0.49)*		–0.34 (–0.62 to –0.06)**		–0.51 (–0.97 to –0.06)**	
	PBF^f^ (%)	–1.04 (–2.44 to 0.35)		–1.11 (–2.30 to 0.08)		0.07 (–1.77 to 1.90)	
	BMR^g^ (kcal/day)	–4.78 (–30.67 to 21.10)		1.44 (–22.61 to 25.49)		–6.23 (–41.48 to 29.02)	
	WHR^h^	–0.06 (–0.17 to 0.05)		–0.02 (–0.03 to 0.00)**		–0.05 (–0.16 to 0.07)	
**MVPA^i^**							
	Weight (kg)	–1.95 (–2.46 to –1.43)*		–0.92 (–1.45 to –0.39)*		–1.03 (–1.76 to –0.29)**	
	TBW (kg)	–0.06 (–0.25 to 0.14)		–0.06 (–0.36 to 0.24)		0.00 (–0.35 to 0.35)	
	Protein (kg)	–0.01 (–0.06 to 0.05)		–0.01 (–0.09 to 0.08)		0.00 (–0.01 to 0.10)	
	Minerals (kg)	0.00 (–0.01 to 0.02)		0.00 (–0.03 to 0.03)		0.01 (–0.03 to 0.04)	
	BFM (kg)	–1.89 (–2.36 to –1.42)*		–0.85 (–1.39 to –0.32)**		–1.03 (–1.74 to –0.33)**	
	FFM (kg)	–0.06 (–0.33 to 0.21)		–0.07 (–0.48 to 0.34)		0.01 (–0.47 to 0.48)	
	SMM (kg)	0.00 (–0.17 to 0.16)		–0.02 (–0.27 to 0.23)		0.01 (–0.28 to 0.31)	
	BMI (kg/m^2^)	–0.76 (–0.95 to –0.57)*		–0.25 (–0.52 to 0.01)		–0.51 (–0.83 to –0.19)*	
	PBF (%)	–1.34 (–1.70 to –0.97)*		–0.57 (–1.01 to –0.13)**		–0.76 (–1.32 to –0.20)**	
	BMR (kcal/day)	–1.18 (–6.93 to 4.57)		–1.51 (–10.37 to 7.34)		0.33 (–9.96 to 10.62)	
	WHR	–0.01 (–0.01 to 0.00)		0.00 (–0.01 to 0.00)		0.00 (–0.01 to 0.00)	

^a^LPA: light physical activity.

^b^TBW: total body water.

^c^BFM: body fat mass.

^d^FFM: fat-free mass.

^e^SMM: skeletal muscle mass.

^f^PBF: percentage of body fat.

^g^BMR: basal metabolic rate.

^h^WHR: waist-to-hip ratio.

^i^MVPA: moderate-vigorous physical activity.

*P<.01.

**P<.05.

## Discussion

### Principal Findings

This study showed that the combined use of a mobile app and a smart band for 3 months, plus brief counselling at the start of the intervention, achieved a slight decrease in weight and BMI but not in other body composition variables. However, subanalyses by BMI, self-reported physical activity, and sex showed a greater decrease in variables such as BMI, WHR, BFM, and PBF in the IG with counselling than in the CG with counselling alone. Adding mHealth as a way of coaching and promoting healthy lifestyles in obese individuals may enhance weight loss outcomes at 3 months. More specifically, the intervention might be more effective in overweight women with moderate physical activity, given that this group experienced greater reductions in weight and body composition variables.

This study offers relevant insight into the effect of mobile apps combined with wearable devices, such as an activity-tracking bracelet, on changing body composition with a large sample size. In recent years, interest in the effects of mHealth on body composition using BIA has increased, giving rise to research such as the TALENT study [39], in which an intensive, web-based lifestyle intervention (Individual Health Management) showed promising results, achieving a mean loss of approximately 10% of the baseline weight and a reduction in BMI, BFM, PBF, and waist circumference at 12 months. Even though the exercise intervention was on a web-based program, and no wearable devices were used, the results are in line with the results of this study. However, the IDEA study [21], in which one of the study groups was provided with wearable technology with a web-based interface for 24 months, did not find differences in body composition. Moreover, the study sample comprised only young adults, and it is not possible to generalize these results to other populations.

Taking studies with mobile apps into account, these results agree with other similar studies assessing the short-term effect of the Noom app (Noom Inc), a commercialized app that provides lifestyle-related logs, mainly food intake and exercise. The Noom app has been studied in combination with human coaching for 8 weeks [40] and alone for 15 and 52 weeks [41], achieving statistically significant decreases in weight, BMI, waist circumference, BFM, and PBF in both sexes. Nonetheless, none of these studies included a proper control group, requiring further research with high-quality methodology, which was the purpose of this study.

In terms of physical activity, a recent meta-analysis [42] demonstrated that a wearable technology intervention duration of more than 12 weeks was significantly more efficient than an intervention of fewer than 12 weeks in terms of BMI outcomes, and a systematic review [43] suggested that an activity tracker combined with a weight loss program may provide superior short-term (less than 6 months) results in middle-aged or older adults. Similarly, an intervention with Fitbit wrist activity tracker (Fitbit Inc) in medical students showed a positive trend for PBF in overweight women and lean body mass in overweight men [44], and another study using an app with push notifications to enhance diet and physical activity showed greater weight loss and body fat loss in obese women [45]. These results are in line with the results of this study, where there was a trend in BFM and PBF to decrease more in the IG, although no significant reduction was observed. In these studies, as in our research, a larger effect on fat mass was observed in women, which may be explained by the influence of psychological determinants, as women are more interested in participating in nutritional interventions [46] because of a desire to lose weight [47]. Also, we obtained a lower participation rate in men than in women, following the trend of the majority of studies of weight management [48], which could have led to body fat differences not being found in the male group in this study. Furthermore, body composition varies depending on sex, as women usually have a larger body fat mass proportion, whereas men are more likely to show greater lean mass, making a larger decrease of fat tissue in interventions with women plausible. Additionally, Slentz et al [49] reported that low amount/moderate-intensity and low-amount/vigorous-intensity endurance training (activity equivalent to 12 miles per week of walking or jogging) were equally effective in reducing the PBF, BFM, and waist circumference in sedentary adults. This result is in line with our findings and may explain part of the improvement in body composition variables in women through increased physical exercise.

The current results show the potential benefit of a short-term mHealth intervention with a mobile app, a smart band, and brief counselling as a useful tool for modifying body composition in overweight and obese healthy people in a primary health context. These findings are clinically relevant for various reasons. First, being an mHealth intervention with no professional face-to-face sessions or follow-up implies that there might be a cost reduction to implementing it in public health programs, and thus, this could be more cost-effective than other approaches. Through this study, we have identified the potential target profile for this intervention: overweight women (aged 18 to 65 years) with moderate physical activity at baseline. However, the physical activity classification was made using the IPAQ, which implies some degree of subjectivity and inaccuracy due to self-reporting. Future studies should explore the classification by accelerometer or another objective source of baseline physical activity in an attempt to obtain similar results. Nevertheless, these findings could be useful for adapting the intervention to population groups, whereby characteristics of each group could be taken into account in increasing the usefulness of the mHealth intervention.

Second, weight and/or BMI cannot solely be used as an accurate indicator of health, since body composition information is also relevant. The analysis of body composition could shed light on this field, allowing us to differentiate between the metabolically healthy but overweight and those with normal weight but with a pathological state. These states can be related to body components (eg, leg fat or SMM), endocrine interactions between individual fat deposits and muscle mass, and/or inflammation [50]. This stratification may be necessary to optimize prevention and treatment strategies and could be measured directly through bioimpedance variables. Furthermore, it is well known that excess body fat and its distribution are associated with metabolic syndrome and insulin resistance [51]. For these reasons, interventions that can modify body composition, focusing on decreasing BFM and PBF and leading to improved health markers, should be a priority in national health policies. In this sense, it is important to implement other measures related to fat distribution in addition to weight or BMI in daily clinical practice for a better approach. Even though our study showed moderate reductions in weight, BFM, and PBF in the IG, current findings support that a reduction in whole-body fat mass could predict changes in cardiometabolic health indices when increasing physical activity [52], even when body weight remains stable. In addition, physical activity seems to have a cardioprotective effect in subjects with higher PBF [53]. Thus, implementing this intervention in daily clinical practice could reduce the cardiovascular risk in overweight and obese people and its associated long-term issues.

Finally, it is important to point out the main limitations of this study. Missing InBody data were greater than expected (123 subjects). The majority of data lost were due to participants not meeting requirements to carry out the measurement, mainly because of medical implant or essential support devices. Despite this fact, losses were similar in both the IG and the CG. Due to the nature of the intervention, this could not be blinded to the participants; however, a recent meta-epidemiological study [54] suggested that blinding is less important than often believed. The duration of the intervention was only 3 months, so we could not measure the sustainability and long-term effect of the intervention. Also, despite the advice provided to subjects at baseline and follow-up visits to avoid the use of other apps related to nutrition or physical activity, we cannot guarantee that other apps were not used.

### Conclusion

The results of this multicenter, randomized clinical trial study showed that, compared with standard counselling alone, using a self-reported app and a smart band obtained beneficial results in weight loss in women and a reduction in BFM and PBF in subjects with a BMI less than 30 kg/m^2^ and moderate-vigorous physical activity level. Further studies are needed to ensure that this profile benefits more than others from this intervention and to investigate modifications of this intervention to achieve a global effect.

## Abbreviations

BFM: body fat mass
BIA: bioelectrical impedance analysis
BMR: basal metabolic rate
FFM: fat-free mass
ICTs: information and communication technologies
IPAQ: International Physical Activity Questionnaire
METs: metabolic equivalents
mHealth: mobile health
PBF: percentage of body fat
PCP: primary care provider
SEEDO: Spanish Society for the Study of Obesity
SMM: skeletal muscle mass
TBW: total body water
WHR: waist-to-hip ratio
